# A review of the genomics of neonatal abstinence syndrome

**DOI:** 10.3389/fgene.2023.1140400

**Published:** 2023-02-10

**Authors:** Elizabeth Yen, Nathan Gaddis, Lauren Jantzie, Jonathan M. Davis

**Affiliations:** ^1^ Department of Pediatrics, Tufts Medical Center, Boston, MA, United States; ^2^ Mother Infant Research Institute, Tufts Medical Center, Boston, MA, United States; ^3^ Tufts University School of Medicine, Boston, MA, United States; ^4^ Research Triangle Institute International, Research Triangle Park, Durham, NC, United States; ^5^ Department of Pediatrics, Johns Hopkins University School of Medicine, Baltimore, MD, United States; ^6^ Tufts Clinical and Translational Sciences Institute, Boston, MA, United States

**Keywords:** neonatal abstinence syndrome (NAS), genetics, epigenetics, inflammation, biomarker

## Abstract

Neonatal abstinence syndrome (NAS) is a constellation of signs of withdrawal occurring after birth following *in utero* exposure to licit or illicit opioids. Despite significant research and public health efforts, NAS remains challenging to diagnose, predict, and manage due to highly variable expression. Biomarker discovery in the field of NAS is crucial for stratifying risk, allocating resources, monitoring longitudinal outcomes, and identifying novel therapeutics. There is considerable interest in identifying important genetic and epigenetic markers of NAS severity and outcome that can guide medical decision making, research efforts, and public policy. A number of recent studies have suggested that genetic and epigenetic changes are associated with NAS severity, including evidence of neurodevelopmental instability. This review will provide an overview of the role of genetics and epigenetics in short and longer-term NAS outcomes. We will also describe novel research efforts using polygenic risk scores for NAS risk stratification and salivary gene expression to understand neurobehavioral modulation. Finally, emerging research focused on neuroinflammation from prenatal opioid exposure may elucidate novel mechanisms that could lead to development of future novel therapeutics.

## 1 Background

From 2010 to 2017, the number of pregnant women with opioid-related diagnoses [e.g., opioid use disorder (OUD)] increased from 3.5 to 8.2 per 1000 delivery hospitalizations (Hirai et al., 2021). This resulted in a concomitant increase in the number of neonates with neonatal abstinence syndrome (NAS) or neonatal opioid withdrawal syndrome (NOWS), from 4.0 to 7.3 per 1000 births^1^. This translated to a healthcare cost of $2.5 billion between 2004 and 2014 (Winkelman et al., 2018). Despite this substantial socioeconomic burden, there remains great variation in the diagnosis and management of NAS, highlighting an urgent need for objective and validated measures to address this challenge.

## 2 Current practice: Clinical evaluation and management of NAS

Multiple factors contribute to the challenge of a diagnosis and management of NAS. These include maternal characteristics (type of opioid used during pregnancy, comorbid physical/mental health conditions), neonatal characteristics (gestational age, sex), and hospital-based practice variation (e.g., location of care, breastmilk, supportive care) (Bogen et al., 2017; Minozzi et al., 2020; Jansson et al., 2017; Favara et al., 2019; Yen et al., 2021; O'Connor et al., 2017; Krans et al., 2021). Clinicians assign the diagnosis of NAS using varying criteria: 1) antenatal opioid exposure, 2) presence of *any* withdrawal signs following birth, and/or 3) use of pharmacotherapy (Jilani et al., 2021). In an effort to address the lack of a standardized clinical definition of NAS, the US Department of Health and Human Services convened a panel that proposed two key elements to diagnose NAS; prenatal exposure of opioids (with/without other substances) and presence of 2 of 5 most common clinical signs of NAS (high-pitched cry, poor sleep, tremors, hypertonia, gastrointestinal issues) (Jilani et al., 2022a; Jilani et al., 2022b).

## 3 Knowledge gaps surrounding NAS

Compounding this diagnostic challenge is lack of objective diagnostic tools. The most commonly used scoring system is the Finnegan Neonatal Abstinence Scoring System (FNASS) (Finnegan et al., 1975). Given the intricacies of the FNASS, attempts have been made to simplify the system with the introduction of multiple approaches including Eat, Sleep, Console (Gomez Pomar et al., 2017; Grossman et al., 2018; Devlin et al., 2020; Kocherlakota et al., 2020). Utilizing other systems such as the NICU Network Neurobehavioral Scale (NNNS) that incorporates neurologic and behavioral measures and signs of stress may provide improved diagnostic information (Lester et al., 2004). Despite the simplification, all scoring systems are subjective and require education/training to minimize inter-rater variability (Timpson et al., 2018; Chin Foo et al., 2021). Scoring systems are tools that may be biased by subjective factors, resulting in over- and under-medication which could actually increase long-term risks.

These scoring systems also do not account for sex of the neonates. While some studies have suggested that male sex is associated with worse NAS, others have not (Jansson et al., 2010; Unger et al., 2011; Charles et al., 2017). Sex is an important biological variable that contributes to the fundamental differences seen in many diseases and pharmacologic responses (Bartz et al., 2020; Kantarci et al., 2020; Zucker and Prendergast, 2020; Sharifi et al., 2021). An urgent need exists to incorporate sex in the evaluation and management of NAS. Based on the current one-size-fits-all model, the field needs molecular and other approaches to arrive at an objective method that considers an individual’s characteristics and enables personalized evaluation and management of NAS.

## 4 Emerging biomarker research in NAS

Researchers have examined molecular mechanisms, genetic and epigenetic alterations that may increase the risk of more severe NAS and the need for pharmacotherapy (which prolongs hospitalizations and increases costs). Several of these approaches are quite promising and have contributed to a better understanding of the pathogenesis of NAS.

### 4.1 Genetics in NAS

#### 4.1.1 Single nucleotide polymorphism (SNP)

Our knowledge of genetic risk factors underlying NAS severity is in its infancy, mainly due to the difficulty of providing sufficient statistical power for genetic analyses. More is known about the genetics of OUD in adults, for which an estimated ∼60% of the population variability is attributable to genetic factors based on twin and family studies (Kendler et al., 2003; Goldman et al., 2005). Early studies of the genetics of NAS consisted primarily of candidate SNP analyses of loci identified in studies of adult opioid dependence or loci associated with opioid and stress pathways, with inconsistent results (Oei et al., 2012; Wachman et al., 2013; Wachman et al., 2015; Mactier et al., 2017; Wachman et al., 2017).

In opioid-exposed neonates, the need for pharmacotherapy was associated with SNPs in cytochrome P450 family 2 subfamily B member 6 (*CYP2B6*). Hypermethylation in the opioid receptor mu 1 (*OPRM1*) promoter was linked to worse NAS outcomes, defined by the need for ≥2 medications (Wachman et al., 2014). Another study reported that SNPs in prepronociceptin (*PNOC*), opioid receptor kappa 1 (*OPRK1*), or opioid receptor delta 1 (*OPRD1*) were linked to the need for ≥2 medications and longer length of hospital stay (Mactier et al., 2017). A recent review provides a comprehensive synopsis of candidate SNP studies and the loci they identified as potentially being associated with NAS severity (Wachman and Farrer, 2019). The results of the candidate SNP studies in are seen in Table 1 and provide an update on the replication status of the identified loci (discussed below). The loci include *OPRM1, OPRK1,* and *OPRD1*, the endogenous opioid peptide *PNOC*, the dopamine-metabolizing enzyme catechol-O-methyltransferase *(COMT)*, and the methadone-metabolizing enzyme *CYP2B6*. The majority of these associations with NAS severity were point-wise significant but failed to reach experiment-wise significance when applying multiple-testing correction.

**TABLE 1:** Variants with evidence of association with NAS phenotypes.

SNP	Gene	Phenotype	Ancestry	N	Study type	Candidate SNP replication (EA^**^, N = 113) [Oei et al. (2012)]	GWAS replication (AA + EA, N = 476) [Bibi et al. (2022)]
rs1799971 [Wachman et al. (2013); Wachman et al. (2015)]	*OPRM1*	LOS	EA^**^	86	Candidate SNP	ND	No
		NAS Treatment					
rs204076 [Wachman et al. (2015)]^*^	*OPRD1 *	LOS	EA^**^	86	Candidate SNP	ND	No
		≥2 Meds					
rs2614095 [Wachman et al. (2015)]^*^	*PNOC*	LOS	EA^**^	86	Candidate SNP	No	No^*^
		≥2 Meds					
rs351776 [Wachman et al. (2015)]^*^	*PNOC *	LOS	EA^**^	86	Candidate SNP	No	No
		≥2 Meds					
rs4732636 [Wachman et al. (2015)]^*^	*PNOC*	LOS	EA^**^	86	Candidate SNP	No	No
		NAS Treatment					
		≥2 Meds					
rs702764 [Wachman et al. (2015)]^*^	*OPRK1*	≥2 Meds	EA^**^	86	Candidate SNP	ND	No
rs4680 [Wachman et al. (2013)]	*COMT*	LOS	EA^**^	86	Candidate SNP	No	No
		NAS Treatment					
		≥2 Meds					
rs740603 [Wachman et al. (2015)]^*^	*COMT*	LOS	EA^**^	86	Candidate SNP	No	No
		NAS Treatment					
rs3745274 [Mactier et al. (2017)]	*CYP2B6*	NAS Treatment	NR	21	Candidate SNP	ND	No
rs2279343 [Mactier et al. (2017)]	*CYP2B6*	NAS Treatment	NR	21	Candidate SNP	ND	ND
rs73313786 [Bibi et al. (2022)]	*SNX13*	NAS Treatment	AA + EA	476	GWAS	ND	ND

NAS, neonatal abstinence syndrome; LOS, length of hospital stay; EA, European ancestry; AA, African ancestry; ND, not done; NR, not reported.

^*^Associations demonstrated point-wise significance at α = 0.05, but not experiment-wise significance.

^**^Cohorts were predominantly EA (98% in the candidate gene discovery cohort (Wachman et al., 2013, Wachman et al., 2015), 88% in the candidate SNP, replication cohort (Wachman et al., 2017)).

#### 4.1.2 Genome-wide association study (GWAS)

For OUD and related phenotypes, GWAS have identified a small number of genome-wide association signals and few replicable findings (Gelernter et al., 2014; Nelson et al., 2016; Polimanti et al., 2020; Song et al., 2020; Zhou et al., 2020; Sanchez-Roige et al., 2021; Gaddis et al., 2022; Kember et al., 2022). Several recent large-scale GWAS of OUD have yielded promising loci with greater statistical support and consistency across multiple cohorts which could provide insight into the genetics of NAS (Zhou et al., 2020; Sanchez-Roige et al., 2021; Gaddis et al., 2022; Kember et al., 2022). A recent NAS GWAS allowed the development of a polygenic risk score (PRS) model that demonstrated the potential of using PRS for predicting NAS severity (Bibi et al., 2022); further model development based on larger sample sizes is needed.

The first ever GWAS examining the need for pharmacotherapy as a measure of NAS severity consisted of 476 *in-utero* opioid-exposed term neonates; 94 were of African ancestry (AA) and 382 European ancestry (EA) (Table 1) (Bibi et al., 2022). Although the sample was small for a GWAS due to the challenge of enrolling *in-utero* opioid-exposed neonates, obtaining consent, and performing genetic testing, this study did identify one genome-wide significant signal on chromosome 7 upstream of the G protein regulator and sorting nexin-13 gene (*SNX13*), which has been associated with heart failure, neutrophil counts, high density lipoprotein cholesterol level, apolipoprotein A1 level, and mean platelet volume (Willer et al., 2013; Li et al., 2014; Chen et al., 2020; Richardson et al., 2020; Vuckovic et al., 2020). *SNX13* has not previously been associated with addiction, but the related *SNX27* has been implicated in attenuating response to cocaine in mice (Munoz and Slesinger, 2014; Rifkin et al., 2018). Given the sample size, it will be important to replicate these findings in an independent dataset. The GWAS failed to replicate any of the associations identified in the candidate SNP studies (Table 1). However, in a gene-based analysis of the GWAS results, *OPRD1* demonstrated some association (*p* = 0.014) as did *PNOC* (*p* = 0.073).

Large-scale adult GWAS of phenotypes related to opioid abuse/dependence have provided greater insight into the genetic loci affecting individual variation in responses to opioids (Table 2). These adult studies have identified 25 independent loci and it will be important to determine whether any of these loci are also associated with NAS severity. Only one locus (*OPRM1*) has been associated with NAS severity, but this finding was not replicated in the NAS GWAS. Of the 25 loci identified in adults, the ones with greatest association with NAS severity in the gene-based analysis performed for the GWAS study are: 1) potassium calcium-activated channel subfamily N member 1 (*KCNN1*) which regulates neuronal excitability and potassium ion trafficking (*p* = 0.00024) (Kember et al., 2022); 2) solute carrier family 2 member 9 (*SLC2A9*) from which is a member of the facilitative glucose transporter family (*p* = 0.035) (Song et al., 2020); and 3) cornichon family AMPA receptor auxiliary protein 3 (*CNIH3*) which has channel regulator activity in the dendritic shaft and postsynaptic membrane and has been associated with schizophrenia (*p* = 0.061) (Drummond et al., 2012; Nelson et al., 2016). Like *KCNN1*, sorting nexins related to *SNX13* (e.g., *SNX14, SNX27*) seem to have a role in regulating neuronal excitability which may cause an attenuated response to cocaine in mice (Huang et al., 2014; Munoz and Slesinger, 2014; Rifkin et al., 2018).

**TABLE 2:** Summary of genome-wide significant loci identified in studies of opioid use disorder and related phenotypes in adults.

Study	Phenotype	Ancestry (N)	Variant-based loci	Gene-based loci
Gelernter et al. (2014)	DSM-IV OD	AA (5,432)	*KCNG2*	NR
		EA (6,877)		
Nelson et al. (2016)	OD	EA (2,637)	*CNIH3*	NR
Polimanti et al. (2020)	DSM-IV OD	AA (7,138)	*C18orf32*	*C18orf32*
Song et al. (2020)	ICD-9/10 OUD	EA (21,310)	*DEFB131*/*SLC2A9*/*RP11–1396O13.13*/*ZNF518B* ^*^	NR
Zhou et al. (2020)	ICD-9/10 OUD	EA (82,707)	*OPRM1*	NR
	DSM-IV OD			
Sanchez-Roige et al. (2021)	POU	EA (132,113)	*KDM4A*/*PTPRF* ^*^; *LRRIQ3*	*KDM4A*; *PTPRF; ARTN*
Gaddis et al. (2022)	OA (DSM-IV OD or FOU-based)	EA (88,114^**^)	*OPRM1*	*OPRM1*; *PPP6C*; *FURIN*
Kember et al. (2022)	ICD-9/10 OUD	AA (88,498)	*NNT*; *CDKAL1*/*SOX4* ^*^; *BTNL2*; *OPRM1*; *MRS2*; *TSNARE1*; *SCAI*/*RABEPK* ^*^; *FBXW4*; *NCAM1*; *FURIN*; *KCNN1*; *RNF114;* chromosome 10 locus	*CHRM2*; *OPRM1*; *FTO*; *DRD2*
		EA (302,585)		
		HA (34,861)		

ICD, international classification of diseases; DSM, diagnostic and statistical manual of mental disorders; OUD, opioid use disorder; OD, opioid dependence; EA, European ancestry; AA, African ancestry; NR, none reported; POU, problematic prescription opioid use; OA, opioid addiction; FOU, frequency of use).

^*^Gene mapping indeterminate.

^**^Effective sample size.

Based on the results of our GWAS on NAS severity, we were able to develop a preliminary set of variants for calculating a PRS for predicting need for NAS treatment (Bibi et al., 2022). The PRS was developed in a training set of 290 EA neonates from the GWAS and it was confirmed to have good predictive ability in independent validation sets of 92 EA and 94 AA neonates when using effect sizes calculated in the validation sets. However, to predict need for NAS treatment in the clinical setting, it is necessary to calculate PRS using the effect sizes from previous analyses, not the neonate being assessed. In this scenario, the PRS did not effectively predict whether a neonate required NAS treatment. While these results demonstrate the potential of PRS for clinical assessment of NAS severity, larger sample sizes are needed to develop more effective PRS models.

#### 4.1.3 Other genetic research in NAS

In a study of 67 neonates (half opioid-exposed), plasma brain-derived neurotrophic factor (*BDNF*) was significantly elevated in opioid-exposed neonates compared to non-exposed neonates, suggesting that plasma *BDNF* might correlate with NAS severity (Subedi et al., 2017). Mahnke et al. (2022) demonstrated that three extracellular microRNAs (miRNAs) in the umbilical cord plasma of opioid-exposed neonates were predictive of NAS severity. In particular, miR-128-3p, miR-30c-5p, and miR-421 predicted the need for pharmacotherapy (area under the curve/AUC of 0.85) and length of hospital stay (AUC 0.90). Adding a few more miRNAs enhanced predictive validity of both models.

Although dopamine receptor type (*DRD2*) is a key reward gene in OUD and has been extensively studied in adults, its role in NAS is not well elucidated. Oei et al. (2012) demonstrated that *DRD2* polymorphisms were detectable in stored blood spots. Furthermore, the ins variant of the −141C Del/Ins polymorphism (located in the promoter region) was more prevalent in non-exposed compared to opioid-exposed neonates who did not require pharmacotherapy. The role of dopamine in NAS is also evident in neonatal salivary transcriptomic studies by Yen et al. (2019). Neonates with NAS have aberrant feeding behavior, especially those with more severe withdrawal. Using drops of saliva, Yen et al. demonstrated that *DRD2* expression was significantly higher in opioid-exposed males than females, evidence of the sex-specific impact of prenatal opioids. Furthermore, a positive correlation was found between *DRD2* expression and feeding volume suggesting that excessive and dysregulated feeding in NAS may be a compensatory mechanism responding to the abrupt end of the opioid supply at birth. The implication of this upregulated reward signaling on future reward-seeking behavior is yet unknown.

### 4.2 Epigenetics of NAS

Although several studies have demonstrated that genetic factors may explain the variability of expression seen in NAS, epigenetic variation in the *OPRM1* gene has also been linked to NAS severity. Cytosine methylation of DNA is a known epigenetic mechanism that results in the addition of a methyl group to cytosine residues of cytosine:guanine (CpG) dinucleotides. Chronic *in utero* opioid exposure may increase methylation at specific CpG sites within promoter regions of a gene, potentially increasing or decreasing gene expression (Nielsen et al., 2012; Doehring et al., 2013). Wachman et al. (2014) obtained DNA from 86 neonates receiving pharmacotherapy for NAS and measured methylation levels at 16 OPRM1 CpG sites. Increased methylation was detected in multiple areas within the OPRM1 promoter, with the highest levels in neonates with the most severe NAS, consistent with gene silencing. In a follow-up study, *OPRM1* methylation was measured in 68 neonates who were exposed to antenatal opioids and their mothers (Wachman et al., 2018). While higher levels at the −18, −14, and +23 CpG sites were associated with receipt of pharmacotherapy in neonates, elevations in mothers were associated with an increased length of neonatal hospital stay, confirming prior findings. In contrast, when placental methylation of the *OPRM1* promoter was analyzed in opioid exposed and non-exposed placentas, no significant associations with neonatal outcome was noted (Wachman et al., 2020).

While these studies suggested that increased methylation of *OPRM1* is associated with more severe NAS, Camerota et al. (2022) hypothesized that pharmacotherapy for NAS would result in decreased DNA methylation and improvements in neonatal neurobehavior. They collected DNA from 37 neonates with NAS before and after treatment and examined methylation at 4 CpG sites within the *OPRM1* gene. Path analysis was used to examine associations with pharmacotherapy, DNA methylation, and NNNS summary scores. DNA methylation did decrease in 1 of 4 CpG sites and was accompanied by less excitability, hypertonia, lethargy, signs of stress and abstinence, and abnormal movement after treatment was completed. Greater decreases in DNA methylation were associated with the most significant decreases in excitability and hypertonia on the NNNS. This study is the first to suggest that pharmacologic treatment in NAS may directly influence epigenetics and neonatal neurobehavior. Further studies are needed in treated and untreated neonates in order to establish a more definitive association.

### 4.3 Genetic evidence of inflammation as a potential mechanism for NAS

Emerging animal data highlight the role of inflammation in potentiating the effects of opioids in drug addiction. Opioids bind with toll-like receptor type 4 (TLR4) and propagate the inflammatory cascade through the release of cytokines and chemokines which precipitate opioid tolerance and dependency (Hutchinson et al., 2012; Wang et al., 2012; Zhang et al., 2020a). There is an urgent need to better understand the impact of inflammation in NAS as it may contribute to the immediate signs of withdrawal and provide the genetic underpinning of the long-term neurodevelopmental effects of antenatal opioids.

#### 4.3.1 Animal studies

TLR4 signaling in the mature central nervous system occurs in response to many types of opioids (e.g., methadone, morphine, buprenorphine, oxycodone) (Jacobsen et al., 2014). Opioids induce a central immune response by binding TLR4 and an accessory protein known as myeloid differentiation protein 2 (MD-2), inducing TLR4 oligomerization and nuclear factor kappa B (NFκB) activation (Wang et al., 2012). This increases the major pro-inflammatory cytokines interleukin 1-beta (IL-1β), tumor necrosis alpha (TNFα), interleukin 6 (IL-6), and monocyte chemoattractant protein-1 (MCP-1/CCL2) and C-X-C motif ligand 1 (CXCL1) (Coller and Hutchinson, 2012). Methadone induces many of these cytokines in the peripheral circulation and brain at P10, approximately human term equivalent (Jantzie et al., 2020). Opioids also activate NFκB, which promotes transcription of opioid receptors and peptides, propagating a detrimental intracellular signaling cascade (Wang et al., 2004; Chen et al., 2007; Rehni et al., 2008; Sawaya et al., 2009; Seney et al., 2021). In adults, microglia have been implicated as a driver for opioid-induced neuroplasticity, catalyzing changes in extracellular matrix, synaptic and dendritic structures, and are proposed to be a cellular bridge connecting opioids to the neural-immune system (Zhang et al., 2020b; Ryu et al., 2021; Seney et al., 2021). Opioid-induced glial activation may serve as an important mechanism underlying drug addiction by opposing opioid analgesia in the mature CNS and enhancing opioid tolerance, dependence, and reward (Hutchinson et al., 2008; Watkins et al., 2009).

Jantzie et al. (2020) demonstrated that opioid-induced brain injuries shared many features of a profound neuroinflammatory disease characterized by the white matter loss and axonal injury seen in non-opioid brain injuries (Newville et al., 2020; Vasan et al., 2021; Madurai et al., 2022). Antenatal opioids primed the immune system with baseline elevations in cytokines/chemokines as well as exaggerated inflammatory responses in peripheral blood mononuclear cells after stimulation with lipopolysaccharide. At term equivalent age, antenatal opioids increased *TLR4* and myeloid differentiation primary response 88 (*MyD88*) mRNA in the fetal brain in conjunction with glial activation, evidenced by increased expression of ionized-calcium binding adaptor molecule 1 (*Iba1*) and changes in microglial morphology and activation. The peripheral inflammation, immune priming, and sustained peripheral immune reactivity (SPIHR) extended beyond the neonatal period (Jantzie et al., 2020; Newville et al., 2020). Even as markers of serum inflammation and SPIHR normalized in adulthood, opioid-induced increases in cerebral immune cell populations (neutrophils and regulatory T-cells) remained (Madurai et al., 2022). Maturing rats with prenatal opioid exposure also had reduced fractional anisotropy (FA) in major white matter tracts, as well as marked loss in cerebral microstructure and abnormalities in directional diffusion. Such robust systemic inflammatory response and immune dysfunction were accompanied by cognitive deficits well into adulthood, evidence of lasting inflammatory impact of antenatal opioids (Jantzie et al., 2020; Madurai et al., 2022).

#### 4.3.2 Neonatal studies

While the impact of prenatal opioids on the developing brain and neurodevelopmental outcomes has been studied in neonates (Yuan et al., 2014; Monnelly et al., 2017; Oei et al., 2017; Sirnes et al., 2017; Merhar et al., 2019; Yeoh et al., 2019; Lowe et al., 2022; Radhakrishnan et al., 2022), the role of inflammation is unknown. Based on the emerging animal data demonstrating the pro-inflammatory effects of antenatal opioids on the offspring, Yen et al. (2022) conducted a novel pilot study to understand the role of inflammation in NAS. Neonatal salivary transcriptomic and brain magnetic resonance imaging (MRI) data demonstrate that opioid-exposed females, regardless of the need for pharmacotherapy, had greater expression of C-C motif chemokine ligand 2 (*CCL2*) and *CXCL1* than males. MRI showed a higher incidence of punctate white matter hyperintensity in opioid-exposed compared to non-exposed neonates, with female predominance. Salivary transcriptomics also showed significantly higher expression of *IL1β, IL6, TNFα,* and *IL10* in opioid-exposed neonates with white matter hyperintensity than in those without it*.* While this pilot study replicated the findings of punctate white matter injury by Merhar et al. (2019), salivary transcriptomic data from this study were the first to suggest the genetic mechanisms by which inflammation may underlie sex-specific white matter injury in opioid-exposed neonates. In a small subset of neonates, opioid-exposed neonates had smaller head circumference and qualitatively reduced FA in major white matter tracts compared to non-exposed neonates (Sikka et al., 2022). This novel research highlights the sex-specific and pro-inflammatory effects of antenatal opioid exposure and supports prior human and animal studies demonstrating the adverse effects of antenatal opioid exposure on brain development at both macro- and micro-structural levels (Monnelly et al., 2017; Jantzie et al., 2020). Genetic underpinning that confirms the role of inflammation in NAS may provide exciting opportunities for non-opioid replacement therapy in neonates most severely affected by the opioid epidemic. Larger sample sizes are needed to validate these findings and correlate them with changes in gene expression.

## 5 Conclusions/future directions

Technology advancement in the last decade has enabled more sophisticated and objective platforms for the diagnosis of NAS, in particular in predicting withdrawal severity and the need for pharmacotherapy. Using cord blood, serum samples, buccal swabs, and drops of saliva, the field progressed rapidly using SNPs, DNA methylation, GWAS, and PRS models that highlights genetic and epigenetic changes resulting from antenatal opioid exposure. Such advancement is key to the systematic understanding and prediction of NAS severity and the discovery of predictive biomarkers that are readily validated. Emerging animal and human data point to the crucial role of antenatal opioids on inflammatory pathways/gene expression in a sex-specific manner. This highlights the potential of non-opioid therapeutics targeting anti-inflammatory pathways. It is important to consider biological variables such as sex and carefully account and adjust for the confounding factors inherent to this type of research. There is also an urgent need to generate larger datasets that validate findings from smaller studies to arrive at robust genetic and epigenetic biomarkers that can be linked to brain structure and function in order to improve neurodevelopmental outcomes of these high-risk neonates.

## References

[B2] Bartz D. Chitnis T. Kaiser U. B. Rich-Edwards J. W. Rexrode K. M. Pennell P. B. (2020). Clinical advances in sex- and gender-informed medicine to improve the health of all: A review. JAMA. Intern. Med. 180, 574–583. 10.1001/jamainternmed.2019.7194 32040165

[B3] Bibi S. Gaddis N. Johnson E. O. Lester B. M. Kraft W. Singh R. (2022). Polygenic risk scores and the need for pharmacotherapy in neonatal abstinence syndrome. Ped. Res. Aug. 16. 10.1038/s41390-022-02243-0 PMC9931940 35974158

[B4] Bogen D. L. Whalen B. L. Kair L. R. Vining M. King B. A. (2017). Wide variation found in care of opioid-exposed newborns. Acad. Pediatr. 17, 374–380. 10.1016/j.acap.2016.10.003 27889436 PMC5420467

[B5] Camerota M. Davis J. M. Dansereau L. M. Oliveira E. L. Padbury J. F. Lester B. M. (2022). Effects of pharmacologic treatment for neonatal abstinence syndrome on dna methylation and neurobehavior: A prospective cohort study. J. Pediatr. 243, 21–26. 10.1016/j.jpeds.2021.12.057 34971656 PMC8960328

[B6] Charles M. K. Cooper W. O. Jansson L. M. Dudley J. Slaughter J. C. Patrick S. W. (2017). Male sex associated with increased risk of neonatal abstinence syndrome. Hosp. Pediatr. 7, 328–334. 10.1542/hpeds.2016-0218 28465360 PMC5519405

[B7] Chen M. H. Raffield L. M. Mousas A. Sakaue S. Huffman J. E. Moscati A. (2020). Trans-ethnic and ancestry-specific blood-cell genetics in 746,667 individuals from 5 global populations. Cell 182 (5), 1198–1213.e14. 10.1016/j.cell.2020.06.045 32888493 PMC7480402

[B8] Chen Y. L. Law P. Y. Loh H. H. (2007). Action of NF-kappaB on the delta opioid receptor gene promoter. Biochem. Biophys. Res. Commun. 352, 818–822. 10.1016/j.bbrc.2006.11.103 17150179

[B9] Chin Foo C. A. Dansereau L. M. Hawes K. Oliveira E. L. Lester B. M. (2021). Improving the assessment of neonatal abstinence syndrome (NAS). Children 8, 685. 10.3390/children8080685 34438576 PMC8394483

[B10] Coller J. K. Hutchinson M. R. (2012). Implications of central immune signaling caused by drugs of abuse: Mechanisms, mediators and new therapeutic approaches for prediction and treatment of drug dependence. Pharmacol. Ther. 134, 219–245. 10.1016/j.pharmthera.2012.01.008 22316499

[B11] Devlin L. A. Breeze J. L. Terrin N. Gomez Pomar E. Bada H. Finnegan L. P. (2020). Association of a simplified Finnegan neonatal abstinence scoring tool with the need for pharmacologic treatment for neonatal abstinence syndrome. JAMA netwk. Open 3, e202275. 10.1001/jamanetworkopen.2020.2275 PMC7142377 32267513

[B12] Doehring A. Oertel B. G. Sittl R. Lotsch J. (2013). Chronic opioid use is associated with increased DNA methylation correlating with increased clinical pain. Pain 154, 15–23. 10.1016/j.pain.2012.06.011 23273101

[B13] Drummond J. B. Simmons M. Haroutunian V. Meador-Woodruff J. H. (2012). Upregulation of cornichon transcripts in the dorsolateral prefrontal cortex in schizophrenia. Neuroreport 23, 1031–1034. 10.1097/WNR.0b013e32835ad229 23103966

[B14] Favara M. T. Carola D. Jensen E. Cook A. Genen L. Dysart K. (2019). Maternal breast milk feeding and length of treatment in infants with neonatal abstinence syndrome. J. Perinatol. 39, 876–882. 10.1038/s41372-019-0374-1 30988400

[B15] Finnegan L. P. Connaughton J. F. Jr Kron R. E. Emich J. P. (1975). Neonatal abstinence syndrome: Assessment and management. Add. Dis. 2, 141–158. (no doi). 1163358

[B16] Gaddis N. Mathur R. Marks J. Zhou L. Quach B. Waldrop A. (2022). Multi-trait genome-wide association study of opioid addiction: OPRM1 and beyond. Sci. Rep. 12, 16873. 10.1038/s41598-022-21003-y 36207451 PMC9546890

[B17] Gelernter J. Kranzler H. R. Sherva R. Koesterer R. Almasy L. Zhao H. (2014). Genome-wide association study of opioid dependence: Multiple associations mapped to calcium and potassium pathways. Biol. Psych. 76, 66–74. 10.1016/j.biopsych.2013.08.034 PMC3992201 24143882

[B20] Goldman D. Oroszi G. Ducci F. (2005). The genetics of addictions: Uncovering the genes. Nat. Rev. Genet. 6, 521–532. 10.1038/nrg1635 15995696

[B21] Gomez Pomar E. Finnegan L. P. Devlin L. Bada H. Concina V. A. Ibonia K. T. (2017). Simplification of the finnegan neonatal abstinence scoring system: Retrospective study of two institutions in the USA. BMJ Open 7, e016176. 10.1136/bmjopen-2017-016176 PMC5623549 28963285

[B22] Grossman M. R. Lipshaw M. J. Osborn R. R. Berkwitt A. K. (2018). A novel approach to assessing infants with neonatal abstinence syndrome. Hosp. Pediatr. 8, 1–6. 10.1542/hpeds.2017-0128 29263121

[B23] Hirai A. H. Ko J. Y. Owens P. L. Stocks C. Patrick S. W. (2021). Neonatal abstinence syndrome and maternal opioid-related diagnoses in the US, 2010-2017. JAMA 325, 146–155. 10.1001/jama.2020.24991 33433576 PMC7804920

[B24] Huang H. S. Yoon B. J. Brooks S. Bakal R. Berrios J. Larsen R. S. (2014). Snx14 regulates neuronal excitability, promotes synaptic transmission, and is imprinted in the brain of mice. PLOS. One 9, e98383. 10.1371/journal.pone.0098383 24859318 PMC4032282

[B25] Hutchinson M. R. Coats B. D. Lewis S. S. Zhang Y. Sprunger D. B. Rezvani N. (2008). Proinflammatory cytokines oppose opioid-induced acute and chronic analgesia. Brain Behav. Immun. 22, 1178–1189. 10.1016/j.bbi.2008.05.004 18599265 PMC2783238

[B26] Hutchinson M. R. Northcutt A. L. Hiranita T. Wang X. Lewis S. S. Thomas J. (2012). Opioid activation of toll-like receptor 4 contributes to drug reinforcement. J. Neurosci. 32, 11187–11200. 10.1523/JNEUROSCI.0684-12.2012 22895704 PMC3454463

[B27] Jacobsen J. H. Watkins L. R. Hutchinson M. R. (2014). Discovery of a novel site of opioid action at the innate immune pattern-recognition receptor TLR4 and its role in addiction. Int. Rev. Neurobiol. 118, 129–163. 10.1016/B978-0-12-801284-0.00006-3 25175864

[B28] Jansson L. M. Dipietro J. A. Elko A. Velez M. (2010). Infant autonomic functioning and neonatal abstinence syndrome. Drug Alcohol Depend. 109, 198–204. 10.1016/j.drugalcdep.2010.01.004 20189732 PMC2875284

[B29] Jansson L. M. Velez M. L. McConnell K. Spencer N. Tuten M. Jones H. (2017). Maternal buprenorphine treatment and infant outcome. Drug Alcohol Depend. 180, 56–61. 10.1016/j.drugalcdep.2017.08.001 28869859 PMC5788458

[B30] Jantzie L. L. Maxwell J. R. Newville J. C. Yellowhair T. R. Kitase Y. Madurai N. (2020). Prenatal opioid exposure: The next neonatal neuroinflammatory disease. Brain Behav. Immun. 84, 45–58. 10.1016/j.bbi.2019.11.007 31765790 PMC7010550

[B31] Jilani S. M. Jones H. E. Davis J. M. (2022). Implementation of a standardized clinical definition of opioid withdrawal in the neonate: Challenges and opportunities. JAMA 327, 1643–1644. 10.1001/jama.2022.5406 35420630

[B32] Jilani S. M. Jones H. E. Grossman M. Jansson L. M. Terplan M. Faherty L. J. (2022). Standardizing the clinical definition of opioid withdrawal in the neonate. J. Pediatr. 243, 33–39.e1. 10.1016/j.jpeds.2021.12.021 34942181

[B33] Jilani S. M. Jordan C. J. Jansson L. M. Davis J. M. (2021). Definitions of neonatal abstinence syndrome in clinical studies of mothers and infants: An expert literature review. J. Perinatol. 41, 1364–1371. 10.1038/s41372-020-00893-8 33514878 PMC8225507

[B34] Kantarci K. Morrow M. M. Miller V. M. (2020). Incorporating sex as a biological variable into clinical and translational research training. J. Womens Health 29, 865–867. 10.1089/jwh.2019.8066 PMC7307681 32208053

[B35] Kember R. L. Vickers-Smith R. Xu H. Toikumo S. Niarchou M. Zhou H. (2022). Cross-ancestry meta-analysis of opioid use disorder uncovers novel loci with predominant effects in brain regions associated with addiction. Nat. Neurosci. 25, 1279–1287. 10.1038/s41593-022-01160-z 36171425 PMC9682545

[B36] Kendler K. S. Jacobson K. C. Prescott C. A. Neale M. C. (2003). Specificity of genetic and environmental risk factors for use and abuse/dependence of cannabis, cocaine, hallucinogens, sedatives, stimulants, and opiates in male twins. Am. J. Psychiatry 160, 687–695. 10.1176/appi.ajp.160.4.687 12668357

[B38] Kocherlakota P. Qian E. C. Patel V. C. Mandru C. Vilar R. E. Alpan G. (2020). A new scoring system for the assessment of neonatal abstinence syndrome. Am. J. Perinatol. 37, 333–340. 10.1055/s-0039-3400310 31777045

[B39] Krans E. E. Kim J. Y. Chen Q. Rothenberger S. D. James A. E. 3rd Kelley D. (2021). Outcomes associated with the use of medications for opioid use disorder during pregnancy. Addiction 116, 3504–3514. 10.1111/add.15582 34033170 PMC8578145

[B40] Lester B. M. Tronick E. Z. Brazelton T. B. (2004). The neonatal intensive care unit network neurobehavioral scale procedures. Pediatrics 113, 641–667. (no doi). 10.1542/peds.113.s2.641 14993524

[B41] Li J. Li C. Zhang D. Shi D. Qi M. Feng J. (2014). SNX13 reduction mediates heart failure through degradative sorting of apoptosis repressor with caspase recruitment domain. Nat. Comm. 5, 5177. 10.1038/ncomms6177 25295779

[B42] Lowe J. R. DiDomenico J. Stephen J. M. Roberts M. H. Rodriguez D. E. Bakhireva L. N. (2022). Early developmental trajectory of children with prenatal alcohol and opioid exposure. Pediatr. Res. Aug 10. 10.1038/s41390-022-02252-z PMC9911560 35948606

[B43] Mactier H. McLaughlin P. Gillis C. Osselton M. D. (2017). Variations in infant *CYP2B6* genotype associated with the need for pharmacological treatment for neonatal abstinence syndrome in infants of methadone-maintained opioid-dependent mothers. Am. J. Perinatol. 34, 918–921. 10.1055/s-0037-1600917 28320034

[B44] Madurai N. K. Kitase Y. Hamimi S. Kirk S. E. Sevensky R. Ramachandra S. (2022). Methadone alters the peripheral inflammatory and central immune landscape following prenatal exposure in rats. Adv. Drug Alcohol Res. 2, 10792. 10.3389/adar.2022.10792 PMC10312988 37396628

[B45] Mahnke A. H. Roberts M. H. Leeman L. Ma X. Bakhireva L. N. Miranda R. C. (2022). Prenatal opioid-exposed infant extracellular miRNA signature obtained at birth predicts severity of neonatal opioid withdrawal syndrome. Sci. Rep. 12, 5941. 10.1038/s41598-022-09793-7 35396369 PMC8993911

[B46] Merhar S. L. Parikh N. A. Braimah A. Poindexter B. B. Tkach J. Kline-Fath B. (2019). White matter injury and structural anomalies in infants with prenatal opioid exposure. Am. J. Neuroradiol. 40, 2161–2165. 10.3174/ajnr.A6282 31624119 PMC6911627

[B47] Minozzi S. Amato L. Jahanfar S. Bellisario C. Ferri M. Davoli M. (2020). Maintenance agonist treatments for opiate-dependent pregnant women. Cochrane Database Syst. Rev. 11, CD006318. 10.1002/14651858.CD006318.pub4 33165953 PMC8094273

[B48] Monnelly V. J. Anblagan D. Quigley A. Cabez M. B. Cooper E. S. Mactier H. (2017). Prenatal methadone exposure is associated with altered neonatal brain development. NeuroImage. Clin. 18, 9–14. 10.1016/j.nicl.2017.12.033 29326869 PMC5760461

[B50] Munoz M. B. Slesinger P. A. (2014). Sorting nexin 27 regulation of G protein-gated inwardly rectifying K⁺ channels attenuates *in vivo* cocaine response. Neuron 82, 659–669. 10.1016/j.neuron.2014.03.011 24811384 PMC4141045

[B52] Nelson E. C. Agrawal A. Heath A. C. Bogdan R. Sherva R. Zhang B. (2016). Evidence of CNIH3 involvement in opioid dependence. Mol. Psych. 21, 608–614. 10.1038/mp.2015.102 PMC4740268 26239289

[B53] Newville J. Maxwell J. R. Kitase Y. Robinson S. Jantzie L. L. (2020). Perinatal opioid exposure primes the peripheral immune system toward hyperreactivity. Front. Pediatr. 8, 272. 10.3389/fped.2020.00272 32670993 PMC7332770

[B54] Nielsen D. A. Utrankar A. Reyes J. A. Simons D. D. Kosten T. R. (2012). Epigenetics of drug abuse: Predisposition or response. Pharmacogenomics 13, 1149–1160. 10.2217/pgs.12.94 22909205 PMC3463407

[B55] O'Connor A. B. Kelly B. K. O'Brien L. M. (2017). Maternal and infant outcomes following third trimester exposure to marijuana in opioid dependent pregnant women maintained on buprenorphine. Drug Alcohol Depend. 180, 200–203. 10.1016/j.drugalcdep.2017.08.012 28917206

[B56] Oei J. L. Melhuish E. Uebel H. Azzam N. Breen C. Burns L. (2017). Neonatal abstinence syndrome and high school performance. Pediatrics 139, e20162651. 10.1542/peds.2016-2651 28093465

[B57] Oei J. L. Xu H. X. Abdel-Latif M. E. Vunnam K. Al-Amry A. Clews S. (2012). Dopamine D2 receptor gene polymorphisms in newborn infants of drug-using women. Arch. Dis. Child. Fetal Neonatal Ed. 97, F193–F198. 10.1136/archdischild-2011-300235 21948329

[B58] Polimanti R. Walters R. K. Johnson E. C. McClintick J. N. Adkins A. E. Adkins D. E. (2020). Leveraging genome-wide data to investigate differences between opioid use vs. opioid dependence in 41,176 individuals from the Psychiatric Genomics Consortium. Mol. Psych. 25, 1673–1687. 10.1038/s41380-020-0677-9 PMC7392789 32099098

[B59] Radhakrishnan R. Vishnubhotla R. V. Guckien Z. Zhao Y. Sokol G. M. Haas D. M. (2022). Thalamocortical functional connectivity in infants with prenatal opioid exposure correlates with severity of neonatal opioid withdrawal syndrome. Neurorad 64, 1649–1659. 10.1007/s00234-022-02939-4 PMC12784300 35410397

[B60] Rehni A. K. Bhateja P. Singh T. G. Singh N. (2008). Nuclear factor-kappa-B inhibitor modulates the development of opioid dependence in a mouse model of naloxone-induced opioid withdrawal syndrome. Behav. Pharmacol. 19, 265–269. 10.1097/FBP.0b013e3282febcd9 18469544

[B61] Richardson T. G. Sanderson E. Palmer T. M. Ala-Korpela M. Ference B. A. Davey Smith G. (2020). Evaluating the relationship between circulating lipoprotein lipids and apolipoproteins with risk of coronary heart disease: A multivariable mendelian randomisation analysis. PLoS. Med. 17, e1003062. 10.1371/journal.pmed.1003062 32203549 PMC7089422

[B62] Rifkin R. A. Huyghe D. Li X. Parakala M. Aisenberg E. Moss S. J. (2018). GIRK currents in VTA dopamine neurons control the sensitivity of mice to cocaine-induced locomotor sensitization. Proc. Natl. Acad. Sci. USA. 115, E9479–E9488. 10.1073/pnas.1807788115 30228121 PMC6176583

[B64] Ryu J. Lee S. Payne B. Gorse K. Lafrenaye A. (2021). Buprenorphine alters microglia and astrocytes acutely following diffuse traumatic brain injury. Sci. Rep. 11, 8620. 10.1038/s41598-021-88030-z 33883663 PMC8060410

[B65] Sanchez-Roige S. Fontanillas P. Jennings M. V. Bianchi S. B. Huang Y. Hatoum A. S. (2021). Genome-wide association study of problematic opioid prescription use in 132,113 23andMe research participants of European ancestry. Mol. Psych. 26, 6209–6217. 10.1038/s41380-021-01335-3 PMC8562028 34728798

[B66] Sawaya B. E. Deshmane S. L. Mukerjee R. Fan S. Khalili K. (2009). TNF alpha production in morphine-treated human neural cells is NF-kappaB-dependent. J. Neuroimmune. Pharmacol. 4, 140–149. 10.1007/s11481-008-9137-z 19023660

[B67] Seney M. L. Kim S. M. Glausier J. R. Hildebrand M. A. Xue X. Zong W. (2021). Transcriptional alterations in dorsolateral prefrontal cortex and nucleus accumbens implicate neuroinflammation and synaptic remodeling in opioid use disorder. Biol. Psych. 90, 550–562. 10.1016/j.biopsych.2021.06.007 PMC8463497 34380600

[B68] Sharifi S. Caracciolo G. Pozzi D. Digiacomo L. Swann J. Daldrup-Link H. E. (2021). The role of sex as a biological variable in the efficacy and toxicity of therapeutic nanomedicine. Adv. Drug Deliv. Rev. 174, 337–347. 10.1016/j.addr.2021.04.028 33957181

[B69] Sikka P. Madan N. Yen E. (2022). Early white matter tract changes in neonates with prenatal opioid exposure: A pilot study. J. Perinatol. Jun 18. 10.1038/s41372-022-01427-0 PMC9759619 35717459

[B70] Sirnes E. Oltedal L. Bartsch H. Eide G. E. Elgen I. B. Aukland S. M. (2017). Brain morphology in school-aged children with prenatal opioid exposure: A structural MRI study. Early Hum. Dev. 106, 33–39. 10.1016/j.earlhumdev.2017.01.009 28187337

[B71] Song W. Kossowsky J. Torous J. Chen C. Y. Huang H. Mukamal K. J. (2020). Genome-wide association analysis of opioid use disorder: A novel approach using clinical data. Drug Alcohol Depend. 217, 108276. 10.1016/j.drugalcdep.2020.108276 32961455 PMC7736461

[B72] Subedi L. Huang H. Pant A. Westgate P. M. Bada H. S. Bauer J. A. (2017). Plasma brain-derived neurotrophic factor levels in newborn infants with neonatal abstinence syndrome. Front. Pediatr. 5, 238. 10.3389/fped.2017.00238 29164087 PMC5675851

[B73] Timpson W. Killoran C. Maranda L. Picarillo A. Bloch-Salisbury E. (2018). A quality improvement initiative to increase scoring consistency and accuracy of the finnegan tool: Challenges in obtaining reliable assessments of drug withdrawal in neonatal abstinence syndrome. Adv. Neonatal Care 18, 70–78. 10.1097/ANC.0000000000000441 29045256 PMC5786483

[B74] Unger A. Jagsch R. Bäwert A. Winklbaur B. Rohrmeister K. Martin P. R. (2011). Are male neonates more vulnerable to neonatal abstinence syndrome than female neonates? Gend. Med. 8, 355–364. 10.1016/j.genm.2011.10.001 22088886 PMC3241965

[B76] Vasan V. Kitase Y. Newville J. C. Robinson S. Gerner G. Burton V. J. (2021). Neonatal opioid exposure: Public health crisis and novel neuroinflammatory disease. Neural. Regen. Res. 16, 430–432. 10.4103/1673-5374.293136 32985461 PMC7996018

[B77] Vuckovic D. Bao E. L. Akbari P. Lareau C. A. Mousas A. Jiang T. (2020). The polygenic and monogenic basis of blood traits and diseases. Cell 182, 1214–1231.e11. 10.1016/j.cell.2020.08.008 32888494 PMC7482360

[B78] Wachman E. M. Farrer L. A. (2019). The genetics and epigenetics of neonatal abstinence syndrome. Semin. Fetal Neonatal Med. 24, 105–110. 10.1016/j.siny.2019.01.002 30709700

[B79] Wachman E. M. Hayes M. J. Brown M. S. Paul J. Harvey-Wilkes K. Terrin N. (2013). Association of OPRM1 and COMT single-nucleotide polymorphisms with hospital length of stay and treatment of neonatal abstinence syndrome. JAMA 309, 1821–1827. 10.1001/jama.2013.3411 23632726 PMC4432911

[B80] Wachman E. M. Hayes M. J. Lester B. M. Terrin N. Brown M. S. Nielsen D. A. (2014). Epigenetic variation in the mu-opioid receptor gene in infants with neonatal abstinence syndrome. J. Pediatr. 165, 472–478. 10.1016/j.jpeds.2014.05.040 24996986 PMC4145036

[B100] Wachman E. M. Hayes M. J. Sherva R. Brown M. S. Davis J. M. Farrer L. A. (2015). Variations in opioid receptor genes in neonatal abstinence syndrom. Drug Alcohol Depend. 155, 253–259. 10.1016/j.drugalcdep.2015.07.001 26233486 PMC4581974

[B81] Wachman E. M. Hayes M. J. Sherva R. Brown M. S. Shrestha H. Logan B. A. (2017). Association of maternal and infant variants in PNOC and COMT genes with neonatal abstinence syndrome severity. Am. J. Addict. 26, 42–49. 10.1111/ajad.12483 27983768 PMC5206487

[B82] Wachman E. M. Hayes M. J. Shrestha H. Nikita F. N. U. Nolin A. Hoyo L. (2018). Epigenetic variation in OPRM1 gene in opioid-exposed mother-infant dyads. Genes Brain Behav. 17, e12476. 10.1111/gbb.12476 29575474

[B83] Wachman E. M. Wang A. Isley B. C. Boateng J. Beierle J. A. Hansbury A. (2020). Placental *OPRM1* DNA methylation and associations with neonatal opioid withdrawal syndrome, a pilot study. Exp. Med. 1, 124–135. 10.37349/emed.2020.00009 PMC7985727 33763662

[B85] Wang X. Douglas S. D. Commons K. G. Pleasure D. E. Lai J. Ho C. (2004). A non-peptide substance P antagonist (CP-96,345) inhibits morphine-induced NF-kappa B promoter activation in human NT2-N neurons. J. Neurosci. Res. 75, 544–553. 10.1002/jnr.10873 14743438

[B86] Wang X. Loram L. C. Ramos K. de Jesus A. J. Thomas J. Cheng K. (2012). Morphine activates neuroinflammation in a manner parallel to endotoxin. Proc. Natl. Acad. Sci. USA. 109, 6325–6330. 10.1073/pnas.1200130109 22474354 PMC3341002

[B87] Watkins L. R. Hutchinson M. R. Rice K. C. Maier S. F. (2009). The "toll" of opioid-induced glial activation: Improving the clinical efficacy of opioids by targeting glia. Trends Pharmacol. Sci. 30, 581–591. 10.1016/j.tips.2009.08.002 19762094 PMC2783351

[B88] Willer C. J. Schmidt E. M. Sengupta S. Peloso G. M. Gustafsson S. Kanoni S. (2013). Discovery and refinement of loci associated with lipid levels. Nat. Gen. 45, 1274–1283. 10.1038/ng.2797 PMC3838666 24097068

[B89] Winkelman T. N. A. Villapiano N. Kozhimannil K. B. Davis M. M. Patrick S. W. (2018). Incidence and costs of neonatal abstinence syndrome among infants with medicaid: 2004**-**2014. Pediatrics 141, e20173520. 10.1542/peds.2017-3520 29572288 PMC5869343

[B90] Yen E. Kaneko-Tarui T. Ruthazer R. Harvey-Wilkes K. Hassaneen M. Maron J. L. (2019). Sex-dependent gene expression in infants with neonatal opioid withdrawal syndrome. J. Pediatr. 214, 60–65. 10.1016/j.jpeds.2019.07.032 31474426 PMC10564583

[B91] Yen E. Madan N. Tarui T. Kaneko-Tarui T. Breeze J. L. Davis J. M. (2022). Sex-specific inflammatory and white matter effects of prenatal opioid exposure: A pilot study. Pediatr. Res. Oct. 24. 10.1038/s41390-022-02357-5 PMC9998341 36280708

[B92] Yen E. Murphy H. J. Friedman H. Lucke A. M. Rodday A. M. (2021). Neonatal abstinence syndrome practice variations across pediatric subspecialty. J. Perinatol. 41, 1512–1514. 10.1038/s41372-020-00831-8 32968221 PMC7985043

[B93] Yeoh S. L. Eastwood J. Wright I. M. Morton R. Melhuish E. Ward M. (2019). Cognitive and motor outcomes of children with prenatal opioid exposure: A systematic review and meta-analysis. JAMA. netwk. Open 2, e197025. 10.1001/jamanetworkopen.2019.7025 PMC6628595 31298718

[B94] Yuan Q. Rubic M. Seah J. Rae C. Wright I. M. Kaltenbach K. (2014). Do maternal opioids reduce neonatal regional brain volumes? A pilot study. J. Perinatol. 34, 909–913. 10.1038/jp.2014.111 24945162

[B95] Zhang H. Largent-Milnes T. M. Vanderah T. W. (2020). Glial neuroimmune signaling in opioid reward. Brain Res. Bull. 155, 102–111. 10.1016/j.brainresbull.2019.11.012 31790721 PMC6946383

[B96] Zhang P. Yang M. Chen C. Liu L. Wei X. Zeng S. (2020). Toll-like receptor 4 (TLR4)/opioid receptor pathway crosstalk and impact on opioid analgesia, immune function, and gastrointestinal motility. Front. Immunol. 11, 1455. 10.3389/fimmu.2020.01455 32733481 PMC7360813

[B97] Zhou H. Rentsch C. T. Cheng Z. Kember R. L. Nunez Y. Z. Sherva R. M. (2020). Association of OPRM1 functional coding variant with opioid use disorder: A genome-wide association study. JAMA. Psych. 77, 1072–1080. 10.1001/jamapsychiatry.2020.1206 PMC7270886 32492095

[B98] Zucker I. Prendergast B. J. (2020). Sex differences in pharmacokinetics predict adverse drug reactions in women. Biol. Sex. Differ. 11, 32. 10.1186/s13293-020-00308-5 32503637 PMC7275616

